# Pharmaceutical supply chain risks: a systematic review

**DOI:** 10.1186/2008-2231-21-69

**Published:** 2013-12-19

**Authors:** Mona Jaberidoost, Shekoufeh Nikfar, Akbar Abdollahiasl, Rassoul Dinarvand

**Affiliations:** 1 Department of Pharmacoeconomics and Pharmaceutical administration, Faculty of Pharmacy, Tehran University of Medical Sciences, Tehran 1417614411, Iran; 2 Dr. Abidi Pharmaceuticals, Abidi Blvd., Tehran 1389776363, Iran; 3 Department of Pharmaceutics, Faculty of Pharmacy, Tehran University of Medical Sciences, Tehran 1417614411, Iran

**Keywords:** Supply chain risks, Pharmaceutical risks, Pharmaceutical supply risks, Medicines supply risks, Risks management

## Abstract

**Introduction:**

Supply of medicine as a strategic product in any health system is a top priority. Pharmaceutical companies, a major player of the drug supply chain, are subject to many risks. These risks disrupt the supply of medicine in many ways such as their quantity and quality and their delivery to the right place and customers and at the right time. Therefore risk identification in the supply process of pharmaceutical companies and mitigate them is highly recommended.

**Objective:**

In this study it is attempted to investigate pharmaceutical supply chain risks with perspective of manufacturing companies.

**Methods:**

Scopus, PubMed, Web of Science bibliographic databases and Google scholar scientific search engines were searched for pharmaceutical supply chain risk management studies with 6 different groups of keywords. All results found by keywords were reviewed and none-relevant articles were excluded by outcome of interests and researcher boundaries of study within 4 steps and through a systematic method.

**Results:**

Nine articles were included in the systematic review and totally 50 main risks based on study outcome of interest extracted which classified in 7 categories. Most of reported risks were related to supply and supplier issues. Organization and strategy issues, financial, logistic, political, market and regulatory issues were in next level of importance.

**Conclusion:**

It was shown that the majority of risks in pharmaceutical supply chain were internal risks due to processes, people and functions mismanagement which could be managed by suitable mitigation strategies.

## Introduction

Access to medicine as a human right is one of the main objectives of healthcare systems [1]. Pharmaceutical supply chain should provide medicines in the right quantity, with the acceptable quality, to the right place and customers, at the right time and with optimum cost to be consistent with health system’s objectives and also it should make benefits for its stockholders [2].

Any risks affecting the pharmaceutical supply chain, not only can waste the resources but also can threaten the patients’ life by hindering access to medicines [3]. Risk management is not only important in the pharmaceutical supply chain, but also is a major player in other aspects of pharmaceuticals such as prescription and uses of medicine [4,5]. Assessing and implementing the strategies to manage the risks in pharmaceutical supply chain is essential in health systems [6]. The importance of the risk management is becoming more vital because medicine is a highly regulated product which is under the controls and tight limitations of public regulatory authorities [7]. Also supply of medicines as strategic goods in developing countries with much economic, social and political instability is faced with more uncertainties and vulnerabilities [8-11].

Supply chain is a set of players, processes, information, and resources which transfers raw materials, and components to finished products or services and delivers them to the customers [12]. It includes suppliers, intermediaries, third-party service providers and customers [13]. It also includes all of the logistics activities, manufacturing operations and activities with and across marketing, sales, product design, finance and information technology [14].

Supply chain management (SCM) is defined as the integration of key business processes across the supply chain for the purpose of creating value for customers and stakeholders [15]. Indeed supply chain management integrates supply and demand within and across companies in an efficient business model [16,17]. The Council of Supply Chain Management Professionals defines supply chain management as planning and management of all activities involved in sourcing, procurement, conversion and all logistics activities [18]. There are various aspects of optimizing in the supply chain; eliminating bottlenecks, balancing between lowest material cost and transportation [19], optimizing manufacturing flow, maintaining the right mix and location of factories and warehouses, vehicle routing analysis, dynamic programming and efficient use of capacities, inventories, and labors are of main aspects of supply chain optimization [20]. All stockholders need to institute the right configuration and adaptability to create best practice and to overcome the obstacles in continues changing environment [21].

Supply chain risk management (SCRM) is a crucial and indivisible part of supply chain managements to achieve mentioned objectives [22]. SCRM attempts to minimize supply chain vulnerability and uncertainties through mitigation plans [23]. Therefore it is essential to identify, assess and prioritize all risks to reduce and control the probability and impacts of unfortunate events [24]. It is aimed to managing the risks in a complex and dynamic supply and demand networks [25].

Various works have been reported regarding different aspects of supply chain risks and risks management in the manufacturing sectors. In pharmaceutical sector, although there are some review studies in supply chain risk management with focus on counterfeit [26-29], supply chain logistics [30], quality assurance [31] and enterprise risk management [32] but there is not any systematic review on the pharmaceutical risk management with perspective of manufacturers risks; meanwhile, there are some systematic reviews on SCRM in other industries [33-35].

This study as a first step of risk management in pharmaceutical supply chain tries to identify all risks which threaten access to medicines through affecting companies supply process as a review.

## Methods

Bibliographic databases such as Scopus, PubMed, Web of Science and scientific search engines such as Google scholar, were searched for pharmaceutical supply chain risk management studies in English language. Searching through databases was done with different keywords: Supply chain management, risk, risk management, risk assessment, pharmaceutical. Searching in each database was adapted to databases characteristics and additionally Medical Subject Headings (MeSH) in searching through PubMed was considered. The last version of searching in mentioned databases carried out in the first week of September 2012.

All results (studies and meeting abstracts) were screened by 4 steps: 1. All results titles were reviewed and none-relevant articles were excluded by outcome of interests and researcher boundaries of study; 2. As some of the articles were found in different databases and also they were duplicated via different groups of keywords, duplicated articles were excluded; 3. After screening the articles, abstracts of all remained articles were reviewed and the none-relevant articles, based on study boundaries, were excluded; 4. In the final step, full texts of all remained articles were read and some of them were excluded by outcome of interest.

The exclusion process was based on consensus of all the authors. Piloted form used for extracting risks from studies.

## Results

### Outcomes of interest

Characteristics of selected articles were summarized in a table and main topic and type of article were recorded. Main topic of papers based on abstract was categorized as follows: supply chain with consumer safety approach, counterfeit, logistic risks, global supply chain, product development risks, environmental risk management, supply chain management with health policy approach, supply chain with company perspective and third parties risk management.

**Figure 1:**
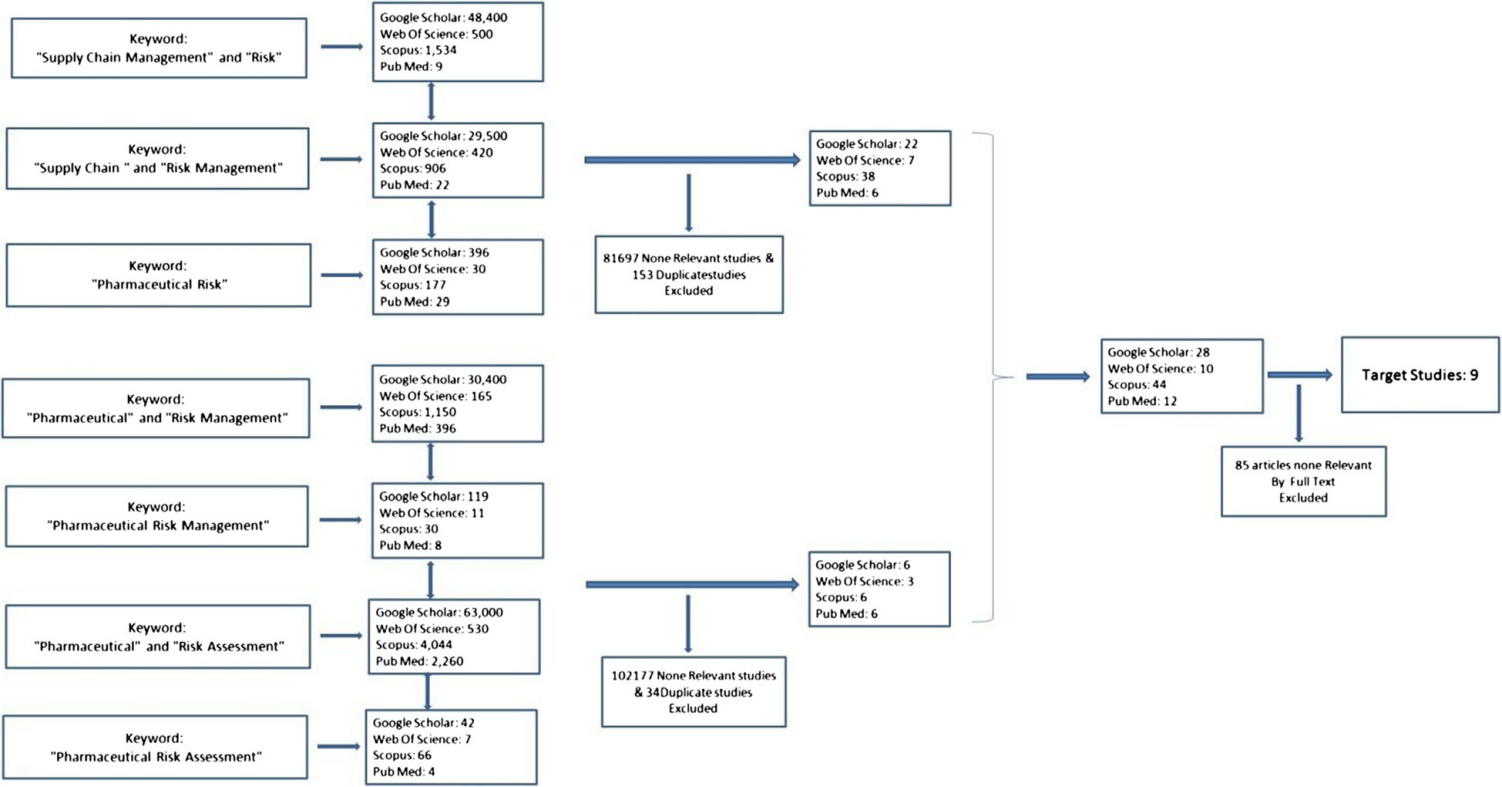
The steps of selecting the articles.

After eliminating repetitious articles and reviewing abstracts, none-relevant articles were excluded and 28 articles from Google scholar, 10 articles from Web of Science, 44 articles from Scopus and 12 articles from PubMed were remained for full text reviewing. Full texts of 94 articles were studied and 85 out of 94 were excluded because they were not adopted with outcomes of interest. Finally 9 articles included in the analysis (Figure 1).

Outcome of interest was defined as pharmaceutical supply chain risk management from production company perspective. It means articles with focus on consumer safety, environmental risk management, health policy and third parties were excluded. After reading full text of 94 articles, 85 out of them were excluded by outcome of interest, design and methodology, and just 9 articles were selected for review study.

Totally, 50 risks were extracted from all selected articles and based on expert opinions were categorized in 7 categories; supply and suppliers issues, organization and strategy issues, financial, logistic, political, market and regulatory issues. Most mentioned risks in articles were related to supply and suppliers. It means 20 out of 50 risks were assigned to supply and supplier issues. 14 risks were related to company organization and strategies, 7 risks were related to financial issues, 3 risks were assigned to market issues, 3 risks were related to political issues, 2 risks were related to logistic issues and 1 item was expressed as regulatory issue.

### Supply and suppliers risks

Supply and suppliers issue was cited in 6 out of 9 articles [8,16,22,36-38]. Partnership with suppliers was mentioned as a risk in 3 [36,37,39]. Ordering cycle time, quality of raw materials and flexibility of supplier were noticed in 2 [36,37]. Contract and agreement issues, customization of suppliers and GMP certificate of supplier mentioned as supply chain risks in 2 [22,36]. Breen has mentioned fragmentation as a risk in pharmaceutical supply chain [22]. Mehralian et al. have mentioned delivery reliability, environmental assessment, technology level, information systems, good will, technology development, flexibility in delivering, flexible quantities, quality management system of supplier and timely delivery as supply risks [36].

### Organization and strategies risks

Inventory management as the most significant risk was mentioned in 4 out of 9 articles [16,22,37,40]. Planning and operation issues, Skill of workers, R&D and Company strategies were reported in three [16,22,37], two [16,41] and two [37,41] respectively. Information flow and visibility on stock were reported in 1 study [22]. In two of articles, organization and process was reported as pharmaceutical supply chain risk [16,37]. Waste management and production cost were demonstrated by Mehralian et al. [36]. Mergers and acquisition was mentioned in one [41] and Time to market and customer service disruption were notified by Blos et al. [37].

### Financial risks

Currency and fluctuation rate were demonstrated in 3 out of 9 articles [8,36,38]. Financial issues were reported in 2 out of 9 articles [36,40]. Tax payable change, costs related to supply, interest rate and tariff policies changes were mentioned in one article [36]. Cash flow was mentioned as a financial risk in another article [22].

### Logistic risks

Counterfeit was considered as logistic risks in 4 out of 9 studies [8,22,38,40]. Transportation risk was reported as pharmaceutical supply chain risk in 3 articles [22,36,37].

### Market issues

Market issues were reported in 2 studies as supply chain risks [22,41]. Consumer taste was mentioned in 2 [16,36] and also demand was reported in 2 out of 9 articles [16,22].

Table 1 shows all risks reported in the selected studies.

**Table 1:** Reported risks with source of studies

**Category**	**Risks**	**A**	**B**	**C**	**D**	**E**	**F**	**G**	**H**	**I**	**Total**
**Supply** & **suppliers issues**	Supply and supplier issue	✓		✓	✓	✓		✓	✓		6
	Partnership with supplier	✓	✓	✓							3
	Raw material quality	✓		✓							2
	Ordering cycle time	✓		✓							2
	Contract & agreements			✓	✓						2
	Customization of supplier			✓	✓						2
	Certificate of GMP			✓	✓						2
	Flexibility of supplier	✓		✓							2
	Fragmentation				✓						1
	Delivery reliability			✓							1
	Environmental assessment			✓							1
	Technology level			✓							1
	Information systems			✓							1
	Good will			✓							1
	Technology development			✓							1
	Flexibility in delivering			✓							1
	Flexible quantities			✓							1
	Flexibility in product variety			✓							1
	Timely delivery			✓							1
	Quality management system			✓							1
**Organization** & **strategies issues**	Customer services disruption	✓									1
	Inventory management	✓			✓			✓		✓	4
	Operation issues	✓			✓			✓			3
	R & D						✓	✓			2
	Skill of workers			✓	✓						2
	Strategy	✓					✓				2
	Planning issues	✓			✓			✓			3
	Information flow				✓						1
	Visibility on stock				✓						1
	Organization & process	✓						✓			2
	Mergers and acquisition						✓				1
	Time to market	✓									1
	waste management			✓							1
	Production cost			✓							1
**Financial**	Tax payable change			✓							1
	Currency rate			✓		✓			✓		3
	Financial risks			✓						✓	2
	Tariff policies changes			✓							1
	Costs related to supply			✓							1
	Cash flow				✓						1
	Interest rate			✓							1
**Logistic**	Counterfeit				✓	✓			✓	✓	4
	Transportation	✓		✓	✓						3
**Market**	Market				✓		✓				2
	Consumers taste			✓				✓			2
	Demand				✓			✓			2
**Political**	Natural disasters & terrorism	✓		✓	✓						3
	Political issues			✓							1
	Sanction			✓							1
**Regulatory**	Regulation		✓		✓	✓	✓		✓	✓	6

**A:** Reference No. 37 (Blos, 2010), **B:** Reference No. 39 (Enyinda, 2009), **C:** Reference No. 36 (Mehralian, 2012), **D:** Reference No. 22 (Breen, 2008), **E:** Reference No. 38 (Enyinda, 2009), **F:** Reference No. 41 (Reschke,2010), **G:** Reference No. 16 (Shah, 2004), **H:** Reference No. 8 (Enyinda, 2010), **I:** Reference No. 40 (Jnandev Kamath, 2012).

## Discussion

It was found that supply and supplier risks were the most important issues discussed in the articles reviewed in this study. It means 40% of risks defined in these studies were related to this category. Regulatory risks were also cited in the majority of articles and it seems that it does have high level of importance in pharmaceutical companies supply chain management but it was not detailed in the studies reviewed in this work. Risks of organization and strategies category are in the next level of importance; because 28% of reported risks were related to this category.

It is the first time that a systematic review in pharmaceutical supply chain risk management with perspective of production companies is carried out; although there are some systematic reviews with focus on logistic, counterfeit, drug safety, quality risk management and etc. in the pharmaceutical industry. For concentrating on the objective of the study and preventing diversity, the keywords were limited by expert opinions. So it could be mentioned as a limitation of study.

No comprehensive study which is exactly matched with outcome of interest in this study was found with selected keywords. Then none of articles which included in this study could cover all aspects of mentioned outcome of interests, and each ones used and covered some parts of supply chain risks in the pharmaceutical companies. Although all authors reached consensus on excluding some studies, but it could be a source for selection bias.

Some of studies about pharmaceutical supply chain risk management in the pharmaceutical companies were published in conferences and published as a conference abstract and unfortunately full text of most of them were not available.

In many of articles just title of risk were found and no detail about them mentioned but some of them reported risks in detail [22,36]. In some of them weigh of risks were measured [8,36,39].

Although in some articles mitigation strategies were discussed; but impact of supply and suppliers risks on whole business and other functions were not mentioned. Then study about impact of risks and investigation about mitigation strategies for each function affected by risks, could be useful.

Mehralian et al., investigated about basic factors involved in risk management of pharmaceutical industry in supplier selection and their priorities. Their study includes literature review, experts’ opinion acquisition, statistical analysis and also using MADM models. They have discussed about 37 factors categorized in 9 main groups. Although just 33 risks of total reported risks are considered in this review. The delivery factor has been identified in this work to have the first priority in the supplier selection and risk management in this regard. Cost and quality are considered in the next level of priorities. The authors used Fuzzy TOPSIS for weighting risks and also discussed about mitigation strategies for mentioned risks in the article. Risks reported in this article, are considered as pharmaceutical supply chain risks but most of them are in supply and supplier selection area [36].

Enyinda et al., investigated about managing risk in pharmaceutical global supply chain outsourcing with applying analytic hierarchy process model. In this study many risks were reported to be related to global supply chain outsourcing but according to scope of our study, just two of reported risks, are considered; regulatory risk and partnership with suppliers. Mitigation strategies were also discussed in this article and Transfer risk is favorable strategy for regulatory risk management [39].

Blos et al. investigated on the external supply chain risk and presented a risk mitigation framework and business continuity plan. In their study around 12 risks related to supply chain risk were reported. Although their study did not exactly assigned to pharmaceutical industry, but risks were mentioned in this article could extend to pharmaceutical supply chain and helpful for risk management process. One of interesting issues in this article is six stages of the business continuity planning process life cycle (risk mitigation management, business impact analysis, supply continuity strategy development, supply continuity plan development, supply continuity plan testing and supply continuity plan maintenance) which could be implement with pharmaceutical supply chain risk management in business units [37].

In “A Preliminary Examination of Risk in the Pharmaceutical Supply Chain (PSC) in the National Health Service (NHS) (UK)”, Breen investigated to identify the nature and prevalence of risk in the pharmaceutical supply chain through workshop forum. Although the perspective of the study was different from our perspective; but as in the paper mentioned, the findings indicated that the risks identified are similar to those prevalent in industrial chains crossing various categories. It was agreed that the top rated risks included fragmentation of the supply chain, lack of visibility concerning placement and availability of stock, inappropriate forecasting conducted by the customer and a general inability of capacity to meet demand [22]. Totally 35 prevalent risks was reported and scored by Breen but according to outcome of interests, just 18 out of them were considered in this study

In “An Empirical Analysis of Risk Mitigation in the Pharmaceutical Industry Supply Chain: A Developing-Country”, Enyinda et al., quantified supply chain risks and prioritized mitigation strategies for managing them through AHP. Food and Drugs Board, supplier failure, counterfeit, currency and exchange rate fluctuation were reported as most important risks in this study. Currency and exchange rate fluctuation were considered as two different risks by Enyinda but in this report both mentioned in the same group [8].

In a work presented at a conference in 2010 by Reschke, he discussed how strategy development and organizational decision-making could affect the course of knowledge growth in order to evaluate potential sources of risks in biopharmaceutical industry. Based on study perspective, markets, regulation, strategy, research and development, mergers and acquisition area were discussed [41].

In another work, Shah studied operational issues in the pharmaceutical supply chain. In this study uncertainty in the demands uncertainty in the pipeline of new drugs, process development, capacity planning, network design and plant design are discussed as some main key issues [16].

Enyinda et al. quantified and prioritized risks which decision makers should consider when deciding on a risk portfolio in “*Quantification of Risk Mitigation in Ghanaian Pharmaceutical Industry Supply Chain”*also the article discussed and ranked mitigation strategies through AHP Model. In this article Food and Drugs Board, supplier failure, counterfeit, currency and exchange rate fluctuation were reported as pharmaceutical supply chain risks. The result of this study was similar with another work reported by the same group [8].

Kamathet al., reported that regulatory risk, financial risk, inventory risk and counterfeit risk are most important issues in pharmaceutical supply chain in India. This study was carried out through a sample population of manufacturers, intermediaries and dispensing pharmacists applying AHP model. Also mitigation strategies ranked and discussed in this article [40].

## Conclusion

Performance of pharmaceutical companies as a main player in pharmaceutical supply chain has significant effect on supply chain management efficiency. Risk identification and mitigating them in pharmaceutical companies not only can lead to process optimization, productivity increase and minimizing business risk, but also will help health systems to meet goals of supply chain management; Accessibility, Quality and Affordability. Many risks reported in this study are internal risks due to processes, people and functions mismanagement in a firm which could be easily managed by suitable mitigation strategies. Although only a few of the risks are external ones but their impact on business disruption have not studied. Therefore identifying their risk impacts of risks on business processes and functions and investigating mitigation strategies to manage them should be considered in future studies.

## Abbreviations

SCRM: Supply chain risk management.

## Competing interests

The authors declare that they have no competing interests.

## Authors’ contributions

MJ conceived and implemented the strategy and methodology, searched through databases, analyzed data and drafted paper, SN gave consultation on designing the study and defining keywords boundaries, reviewed and commented on the manuscript, AA gave consultation on designing the method, defining outcome of interests and selecting articles. RD conceived the strategy of study and supervised the methodology and selecting studies. All authors read and approved the final manuscript.
